# Associations between Problematic Gambling, Gaming, and Internet Use: A Cross-Sectional Population Survey

**DOI:** 10.1155/2019/1464858

**Published:** 2019-09-24

**Authors:** J. Karlsson, N. Broman, A. Håkansson

**Affiliations:** Lund University, Faculty of Medicine, Department of Clinical Sciences Lund, Psychiatry, Lund, Sweden

## Abstract

**Background:**

While pathological gambling, or gambling disorder, is an established diagnosis, a link to other potential behavioural addictions has been suggested. The present study aimed to investigate whether signs of problem gaming and problematic internet use are related to problem gambling in the general population, while including other potential risk factors.

**Methods:**

A cross-sectional study design, using an electronical questionnaire, administered through a marketing survey company for relative representativeness with respect to age and gender. Potential correlates of problem gambling were measured in binary analyses, and significant associations were entered in a logistic regression analysis controlling them for one another. Problem gambling, gaming, and internet use were measured through established screening instruments (the CLiP, the GAS, and the PRIUSS).

**Results:**

Statistically significant associations were found between problem gambling and both problem gaming and problematic internet use, as well as with male gender. In logistic regression, problem gaming, problematic internet use, and male gender remained associated with problem gambling.

**Conclusion:**

After controlling for potential demographic risk factors, problem gaming and problematic internet use may be related to problem gambling, suggesting that these constructs may interact or may share similar risk factors. More research is needed to clarify factors mediating the links between these conditions.

## 1. Introduction

Gambling for money is a global phenomenon with a long history in human societies. Severe forms of gambling behaviour are diagnosed as gambling disorder, a persistent and recurrent maladaptive gambling behaviour, which leads to clinically significant impairment and distress [1–4]. The concept of problem gambling, including both a diagnosed gambling disorder and a subdiagnostic level of problematic gambling, has been estimated to have prevalence between 0.1 and 5.8% across continents [5]. Altogether, problematic gambling is known to be associated with significant mental health problems [6, 7] and with an increased risk of suicidal behaviour [8] and suicide death [9].

Gambling disorder was the first behavioural addiction recognized as a disorder in the same category as alcohol- and drug-related conditions. In contrast to the relatively well-established concept of gambling as a phenomenon causing addiction [10], other nonsubstance-related conditions are discussed as potential separate diagnoses, including the concept of problematic video gaming or internet use. Here, the DSM-5 manual included internet gaming disorder as a “condition for further studies” [10], and recently, this condition (named gaming disorder) was recognized as a disorder by the World Health Organization [11]. Addiction to gaming has been described to be associated with consequences related to preoccupation, extensive time investment, lack of priority given to other activities, and even health-related harms [12]. Aside from the gaming disorder diagnosis, another tentative diagnosis, an internet addiction diagnosis, was under evaluation for inclusion into the DSM-5, but hitherto it has not been recognized as a diagnostic entity, although widely recognized as a clinical and public health issue [13–16].

Intuitively, it is of interest to examine to what extent these nonsubstance-related addictive behaviours may be related to one another. Where a potential link between problem gaming and problem gambling has been examined, results have not been consistent; some data have indicated an association between gambling and gaming [17, 18], whereas other studies have seen important differences [19], including differences between the characteristics of patients with gaming and gambling problems, respectively [20]. Theoretically, a potential link between gambling and addictive behaviours evolving online may be of particular interest in settings where a large proportion of gambling is carried out online. In the geographical setting studied here, online gambling is commonly reported in the treatment setting, to the extent that a large majority of treatment seekers [21] and helpline callers [22] report online gambling as their problematic gambling type. A problematic gambling pattern may be particularly likely to develop in gambling types carried out online [23, 24], and it has been described that online gambling may be associated with higher rates of mental illness, psychological distress, or alcohol consumption, compared to land-based forms of gambling [25–28].

Thus, problem gambling may be associated with online behaviour, and the tentative diagnostic construct of internet addiction has been cited as a risk factor for problem gambling [29]. Also, authors have suggested that social casino gambling, commonly appearing in social media, may represent a risk factor of problem gambling [30], such that users of these social casino gambling services may migrate to online gambling for money [31], and that these behaviours may be interrelated [32]. Altogether, problem gambling may be more likely in individuals with a problematic or addictive online or gaming behaviour, although these associations are so far not conclusive and require more research [33, 34]. However, several other risk factors of problem gambling are known. Studies have linked problem gambling with male gender [29, 35, 36], although women might have elevated risk of problem gambling in relation to gambling online [28], and problem gambling also has been associated with a lower level of education [36, 37]. Regarding both employment and age, findings have been diverse, as both being employed [2, 38] and unemployed [36] have been found to be associated with problem gambling in different studies, and problem gambling has been shown to be linked to older age in general [38], younger age in men, and older age in women [36]. Homosexual and bisexual orientation has also been suggested to be associated with problem gambling [39], although a more recent study failed to demonstrate this association [40].

Based on the hitherto uncertain association of problem gambling with other behavioural addictions, such as problematic gaming and internet use, the present study aimed to investigate whether problem gambling in a general population survey may be associated with these two factors, when controlling for a number of known or likely risk factors of problem gambling, such as age, gender, sexual orientation, mental health, occupation, and social isolation.

## 2. Materials and Methods

### 2.1. Study Design and Population

An online survey was distributed in Sweden through a marketing survey company. The questionnaire was designed as a self-reporting test. Participants were to be above 15 years of age at the time of the study (in the present setting, individuals aged 15 years and above can independently consent to study participation, provided this is approved by a regional ethics board). The study used an online questionnaire, which was designed by the company GHM Patient Information Broker AB and distributed electronically to a web panel provided by the company Userneeds (https://userneeds.com/en/). Userneeds is a company operating in several European countries, and participants have voluntarily registered with this company and agreed to receive different types of surveys, such as market surveys and similar. The present type of web panel has previously been used in other research studies in the area [41]. For participants in the Userneeds web panel, a monetary compensation is provided, with credits corresponding to a value of around one euro for every survey taken by the participant. In the present study, Userneeds web panel members in Sweden were addressed, until an equal proportion of women and men was obtained and until a satisfactory proportion of age groups, corresponding to the general population, was reached, with the aim of reaching 1,500 complete surveys in a sample being roughly representative of the Swedish population with respect to gender and age. Surveys from a total of 1,860 individuals were opened. Among them, 1,593 surveys were considered complete, with full data for the key variables included, i.e., the data describing gambling, gaming, and internet use, and these individuals were finally included. The data collection was carried out for 15 days in December 2017. Individuals participating in the Userneeds web panel received the survey, under the headline “Are you addicted to gambling or to the web? Got control of your gambling behaviour? A self-test about disordered gambling and internet behaviour for all those aged 15 years or above. Test yourself!” The information following that headline gave instruction about the study, including the fact that questions were to address gambling, gaming, and internet use, as well as social contact and well-being, that data in the study would remain strictly confidential and that the researchers would be unaware of the identity of the respondents. The survey was opened only if the respondent chose to provide informed consent to the study. Because of the study design, information on national helplines and professional help was included and recommended for subjects scoring above cutoffs for each test, and subjects were informed about this in the study information.

Ethical approval was obtained from the Regional Ethical Review Board in Lund, Sweden (file number 2017/791).

### 2.2. Measures

For the screening for problem gambling, the CliP (Loss of Control, Lying, and Preoccupation) was used. It is based on three items and has shown sufficient accuracy in screening for problem gambling, where one or more affirmative answers are coded as a positive problem gambling screen [42]. For problem gaming, the Game Addiction Scale (GAS), developed by Lemmens and coworkers, was used. It includes seven questions regarding gaming behaviour and constitutes a shortened version of the original 21-item test and has shown good reliability and validity [43–45]. For problematic internet use, the 3-item PRIUSS screening test was used. It is a shortened version of the instrument PRIUSS-18 and has been tested and confirmed as an appropriate screening instrument for problematic internet use [46], where the lowest score is 0 and the highest is 12.

For gambling, this cutoff was set at the established level of one affirmative answer or more [42], and for the GAS, the scoring of 3 (on a 1–5 Likert scale, where 3 represents a study item to occur at least “sometimes”) on at least four of the seven items was used as a cutoff [43]. For the PRIUSS, although less established, a score of six or more was chosen as the cutoff for providing a recommendation at the end of the survey, in order to set the level conservatively at the threshold level with the highest specificity as reported in the original publication [46]. For all screening tools included, the previously translated versions used in a pilot study on online behaviour preceding the present study [40] were used. Back translation into English was carried out by an independent person with native level of Swedish and English and judged to yield satisfactory results. Internal consistency was calculated for the three scales measuring problem gambling, problem gaming, and problematic internet use, with Cronbach's alpha values of 0.68 for the CLiP, 0.89 for the GAS, and 0.81 for the PRIUSS.

Other variables that were recorded were age, gender, sexual orientation, primary occupation, number of friends outside the internet (too many, satisfactory number, or too few), and whether the individual had ever felt a need to seek treatment for psychological distress. Sexual orientation was coded into heterosexual and nonheterosexual. Occupation was dichotomized into having an occupation (student and employed) vs no occupation (unemployed, retired, and others). Age was divided into categories, ranging from 15–19 years of age to 60 or above (Table 1). Having thought of seeking help for psychological distress had the option to not reply, and these answers were excluded from the analysis. For the number of friends outside of the internet, data were dichotomized as satisfactory or too high number of friends, vs too few.

### 2.3. Statistical Analyses

In SPSS, respondents with problem gambling and respondents without problem gambling were compared in bivariate analyses, using the chi-square test for categorical variables and the Mann–Whitney *U* test for the comparison of GAS and PRIUSS data. For descriptive purposes, percentages were reported for categorical variables, and mean and standard deviations (SD), as well as medians and interquartile ranges, were reported for the scoring on GAS and the PRIUSS. Thereafter, all variables were entered into a logistic regression with problem gambling as the dependent variable, controlling independent variables for one another. In the regression analysis, 37 individuals with missing data for demographic variables were excluded, leaving a final sample of 1,556 individuals in the logistic regression. Associations with a *p* value less than 0.05 were considered significant, and the logistic regression model was analysed with odds ratios with 95% confidence intervals (CI) for each of the potential correlates of problem gambling.

## 3. Results

Sample characteristics are displayed in Table 1, including data for problem gambling, problem gaming, and problematic internet use. Based on the CLiP, 90.2% scored 0 (no problem gambling) and 9.8% scored 1 to 3 (problem gambling). There was a significant relationship between problem gambling and gender (*p* < 0.00001) but not between problem gambling and sexual orientation (*p*=0.82), age (*p*=0.71), having enough friends outside of the internet (*p*=0.87), having considered seeking help for psychological distress (*p*=0.06), or occupation (*p*=0.30). GAS and PRIUSS scores were significantly associated with problem gambling (*p* < 0.001, Table 2).

In logistic regression (Table 3), male gender, a higher degree of problem gaming (GAS), a higher degree of problem internet use (PRIUSS), and having enough friends outside the internet remained significantly and positively associated with problem gambling.

## 4. Discussion

The present study, focusing on correlates of problem gambling in the general population, demonstrated that both problem gaming scores and scores on problematic internet use were significantly associated with problem gambling. These associations remained when controlling these conditions for one another and when controlling for other potential risk factors for problem gambling, including gender.

In total, this lends support to the hypothesis of an association between these conditions. It has been reported that correlates of video game addiction and social media addiction share similarities with problem gambling, such as increased odds of ADHD and lower education [47], and that internet addiction may be associated with problem gambling [29], whereas in contrast, it also has been reported that the association between gambling and gaming may be weak or only the result of an overlap of risk factors [33, 34]. Although the problem patterns studied here seem to be associated with one another, the mechanisms driving each of these problem behaviours are likely to be diverse. Gambling for money is known to be driven by the reinforcing properties of a game involving a financial risk-taking related to a particular event which may result in an instant monetary reward, and the evolution of addiction involves a maladaptive pattern of handling impulses to gamble and erroneous beliefs about the chance of winning. In contrast, an addictive behaviour related to gaming typically lacks the immediate award-winning component but involves slower reinforcing components provided from the progression and the “levelling up” within the game. Thus, even when components in video gaming may mimic gambling, the progress into each type of addiction is likely to be diverse [48]. This is supported by the research indicating that problem gamers and problem gamblers differ with respect to a number of characteristics [20]. Likewise, for problematic internet use, a concept corresponding to the narrower concept of internet addiction, it has been argued that the mechanisms behind this concept are likely to differ depending on the content of online overuse, rather than as a separate addictive behaviour itself [49], and that an addiction-like use of the internet may be associated with different types of computer-mediated maladaptive behaviours, including problem gaming [50]. Altogether, although problem gambling, problem gaming, and problematic internet use may represent different maladaptive behaviours with diverse explanatory mechanisms, the present cross-sectional study in a general population-based web panel indicates that an association between these types of addictive behaviours is likely. This supports, for example, the active screening for the other behaviours in patients seeking treatment for one of them.

The present study focused on whether problem gaming and problematic internet use were associated with problem gambling, while controlling for a number of other potential risk factors. In this adjusted analysis, male gender was associated with problem gambling, consistent with previous research [35] (although in contrast, a study from the present setting demonstrated a higher or similar prevalence of problem gambling in adolescent females [51]). Thus, the gender association with problem gambling in the present study was altogether unsurprising. With respect to sexual orientation, described in one previous report as a potential risk factor [39], no significant association with problem gambling was seen. This finding is consistent with a smaller pilot study from the present setting, which failed to demonstrate an increase in problem gambling in sexual minorities [40].

Having enough friends outside the internet was positively associated with problem gambling. This finding may seem surprising; although this particular topic has not previously been addressed to a large extent, one study showed that problem gambling was associated with feelings of loneliness [52]. Also, the present finding may seem contradictory to the gambling patterns of the present setting, where a majority of patients seeking help for a gambling disorder have a predominant gambling pattern on the internet [21], which typically would not occur in the context of social relations. As gambling patterns change towards an increasing proportion of online gambling, associations between social integration and problem gambling may become increasingly complex, and future studies may be needed in order to highlight associations between problem gambling and relationship factors and how these factors apply to modern gambling patterns.

Employment was unrelated to problem gambling. This may also be a surprising finding, but studies have not been conclusive as to whether employment is associated or not with problem gambling. For example, one literature review found problem gambling to be linked with higher income [5], in population surveys in Estonia [53] and Italy [54]. Also, blue-collar work [36] and being employed for wages [2] are factors that have been associated with problem gambling. Variables describing whether an individual is employed or studying may be using too broad definitions to capture reasons behind excessive gambling, and more studies may need to address associations with occupational status in a narrower way than what could be achieved in the web survey design used here.

In the present study, reporting of psychological distress to a level where an individual considers seeking help failed to demonstrate a significant and independent association with problem gambling in the adjusted analysis. This may be seen as surprising; studies in the field have shown associations of mental illness and problem gambling [2, 25]. In contrast, for the gambling problem itself, it is well known that many people do not seek treatment because of a range of different barriers to treatment seeking [55, 56], including barriers perceived by concerned significant others [57]. Also, despite high psychiatric comorbidity in problem gambling, mental health issues have not necessarily been reported to represent the major motivator behind readiness to seek treatment; rather, financial problems and relationship issues are commonly reported to drive treatment-seeking behaviour [58]. In the present study, problem gamblers were not significantly more likely to report a history of feeling a need to seek treatment for psychological problems. Although this formally does not address only the actual treatment-seeking behaviour but also the feeling of a need to obtain treatment, it is possible, based on previous literature, that many problem gamblers may not readily identify as people in need for formal treatment [55]. Also, in the present setting, formal treatment options for the gambling problem itself largely have been lacking, with an overall very low treatment coverage for problem gambling [9]. Although this is currently changing [21], many people with a problematic gambling pattern may still perceive barriers to identifying as individuals in need for treatment. Here, future studies are needed in order to highlight how the perception of treatment needs changes in different settings depending on organizational and legislative changes in the provision of formal treatment or other types of support.

The present work has a number of potential limitations. Due to the cross-sectional study design, the results indicating associations between internet, gaming, and problem gambling do not imply causal associations, and longitudinal studies are needed in order to fully understand the interplay between problem gambling and other potentially addictive nonsubstance-related behaviours over time. Also, the CLiP instrument does not differentiate previous and current problem gambling, whereas the GAS assesses past-6-month symptoms, and the PRIUSS addresses current internet use, making it more difficult to assess the temporal order of different issues studied here. In addition, it should be borne in mind that the three instruments used for this study are shortened versions of instruments, intended for problem screening, and in this relatively novel area of research, cutoff values are sparsely addressed in previous literature, such that associations reported here can lead to assumptions about risk behaviours and not about actual diagnostic associations. In addition, the present paper included a sample of individuals who were voluntarily included in a web panel of a market survey provider, possibly with the implication that people included may theoretically have a higher online involvement and a higher interest in gambling issues. Importantly, the rate of problem gambling, measured with the NODS-CLiP [42] screening for a lifetime history of problem gambling, was higher than in the range of studies demonstrating prevalence measures of problem gambling as measured with more extensive assessment instruments than the brief tool used here [5]. However, this proportion was very close to that seen in the pilot study by Broman and Håkansson [40], which was designed as a self-selected web survey with the same screening tool as the one used here, and slightly higher than in another self-selected survey addressing gambling and related issues (8.1 percent [59]), thus comparable to other studies including primarily individuals with a high degree of online involvement. For comparison, a US general population survey conducted by telephone, and using the same screening instrument as here, identified only 3.3 percent problem gamblers [60], whereas another telephone survey using the same instrument, but in a military cohort hypothesized to represent a potential high-risk group, revealed eight percent problem gambling [61]. Thus, the proportion of problem gamblers in the present study was high but possibly related to the online recruitment and comparable to other similar studies. In addition, a prevalence estimate was not the primary aim of the present study where associations with demographic and other addictive variables were instead the main focus.

As gambling, at least in the present setting, increasingly happens online, it cannot be excluded that parts of the problematic internet use perceived in people with extensive gambling behaviours may in fact cause an overlap between these two potential nonsubstance addictions. For example, online casino gambling is reported as the problematic gambling type by a majority of treatment seekers in the present setting [21], such that one part of these individuals' internet use may represent the gambling behaviour. In addition, it has been reported in recent years that gambling and gaming in adolescents may converge through the introduction of betting components within video games [62]. However, the items addressing gambling in the study do not specifically address internet gambling, and likewise, the screening items for problematic internet use do not address gambling. While the present study setting has a very high rate of online gambling, particularly among treatment seekers [21], this limitation may apply to a larger extent than in countries where online gambling composes a smaller proportion of gambling or problem gambling, as in several settings, including Spain, South Africa, Australia, and the US, considerably lower rates of online gambling have been reported in treatment-seeking gamblers or helpline callers [63–66]. Thus, although separate nonsubstance-related addictive behaviours may be associated with one another and may share overlapping risk factors, study procedures are not likely to explain this to a large extent.

The instruments used in the study were translated into Swedish for the first time in previous studies and used with the same wording here [40]. Although a back translation through an independent translator was carried out with satisfactory results, it cannot be excluded that minor alterations in how questions are perceived by respondents may change the percentage of people endorsing a particular item, compared to the original English version. However, as the instruments were not intended to provide a prevalence measure in comparison to other settings but rather to assess associations within a same language sample studied in the present setting, this may have a limited influence on the findings of the present study.

Strengths of the present study, however, may include the anonymous reporting of data used here and the relative representativeness with respect to age and gender in the present dataset. In addition, the present study includes three nonsubstance-related addictive behaviours, only one of which is an established diagnosis, and a brief population-based screen may provide more information about associations of behaviours than would a diagnostic assessment in a narrower, clinical setting. This, while brief screening tools lose in the exactness of information, they provide behaviour data in a broader sense and may improve understanding of these behavioural addictions in an early phase of research development.

## 5. Conclusions

The present study concludes that there is a positive relationship of symptoms of problem gaming and problematic internet use, respectively, with problem gambling, even when controlling for one another and when controlling for male gender representing a well-known risk factor. While more research is needed in larger and more in-depth studies, including in settings with a less pronounced online gambling pattern, these findings suggest that further attention should be paid to problematic behaviours in video gaming and in behaviours indicating an exaggerated use on online services.

## Figures and Tables

**Table 1:** Group characteristics (*N* = 1,593).

	% (*n*)
Age	
15–18 yrs	4 (66)
19–24 yrs	11 (179)
25–29 yrs	10 (158)
30–39 yrs	18 (282)
40–49 yrs	18 (291)
50–59 yrs	19 (300)
60+ yrs	19 (310)
Missing	0 (7)

Gender	
Male	49 (783)
Female	48 (772)
Transgender	0 (2)
Missing	2 (36)

Sexual orientation	
Heterosexual	93 (1,483)
Homosexual	2 (24)
Bisexual	4 (63)
Others	1 (22)
Missing	0 (1)

Occupation	
Employed	65 (1,043)
Studying	14 (222)
Job-seeking	4 (58)
Retired	14 (220)
Others	3 (49)
Missing	0 (1)

Number of friends outside the internet	
Satisfactory	76 (1,207)
Too many	5 (72)
Too few, feeling lonely	20 (314)

Ever sought treatment for psychological distress	
Yes	31 (489)
No	67 (1,061)
Prefer not to answer	3 (43)

Gambling (CLiP, number of items)	
0	90 (1,437)
1	6 (98)
2	2 (35)
3	1 (23)

Gaming (GAS score)	Mean 9.65 (std dev 3.94), median 8 (IQR 7–11)

Internet use (PRIUSS score)	Mean 2.42 (std dev 2.43), median 2 (IQR 2–4)

**Table 2:** Comparison between problem gamblers and other individuals.

	Problem gambling (CLiP > 0, *n* = 156)	Nonproblem gambling (CLiP = 0, *n* = 1,437)	*p* value	Missing
Age groups			0.71	7
15–18 yrs	3% (*n* = 4)	4% (*n* = 62)		
19–24 yrs	13% (*n* = 20)	11% (*n* = 159)		
25–29 yrs	9% (*n* = 14)	10% (*n* = 144)		
30–39 yrs	19% (*n* = 29)	18% (*n* = 253)		
40–49 yrs	22% (*n* = 34)	18% (*n* = 257)		
50–59 yrs	17% (*n* = 26)	19% (*n* = 274)		
60+ yrs	18% (*n* = 28)	20% (*n* = 282)		
Male gender	68% (*n* = 105)	48% (*n* = 678)	<0.00001	36
Heterosexual	94% (*n* = 146)	93% (*n* = 1,337)	0.82	1
Occupation (employed or student)	76% (*n* = 119)	80% (*n* = 1,146)	0.30	1
Enough friends outside the internet	81% (*n* = 126)	80% (*n* = 1,153)	0.87	0
Ever sought treatment for psychological distress	37% (*n* = 58)	30% (*n* = 431)	0.06	0
GAS score	12.60	9.33	<0.001	0
PRIUSS score	3.62	2.30	<0.001	0

Statistical associations were calculated with the chi-square test for categorical variables and the Mann–Whitney *U* test for continuous variables.

**Table 3:** Associations with problem gambling: logistic regression.

	OR	95% confidence interval
Older age group	1.01	0.99–1.02
Occupation	0.75	0.47–1.20
Heterosexual	1.36	0.65–2.82
Enough friends outside the internet	1.70	1.05–2.74^*∗*^
Male gender	2.89	1.92–4.34^*∗*^
Ever sought help for psychological distress	1.30	0.86–1.96
Problem gaming (GAS)	1.14	1.09–1.19^*∗*^
Problem internet use (PRIUSS)	1.16	1.07–1.26^*∗*^

All individuals without missing data are included (*N* = 1,556) (^*∗*^significant associations).

## Data Availability

Data can be made available upon request.
